# The Role of Bandage Contact Lenses Versus Amniotic Membrane in Corneal Wound Healing: A Systematic Review

**DOI:** 10.7759/cureus.87260

**Published:** 2025-07-04

**Authors:** Ali Zain Abden M AlShammari, Zohair Khouqeer, Safaa Medani, Zeinab Elbashir Abdelgadir, Zayed Alnefaie

**Affiliations:** 1 College of Medicine, Al-Rayan Colleges, Medinah, SAU; 2 Department of Ophthalmology, King Faisal Specialist Hospital and Research Centre, Medinah, SAU; 3 Department of Ophthalmology, Al Rayan National College of Medicine, Medinah, SAU; 4 Department of Anatomy and Embryology, Al-Rayan Colleges, Medinah, SAU

**Keywords:** amniotic membrane, bandage contact lenses, corneal ulcer, epithelialization, wound healing

## Abstract

Corneal wound healing plays a vital role in restoring ocular surface integrity following injury or disease. Both bandage contact lenses (BCLs) and amniotic membranes (AM) are non-pharmacologic interventions widely used in ocular surface disease management. This systematic review focuses on presenting high-level clinical evidence supporting the effectiveness of bandage contact lenses versus amniotic membrane in corneal wound healing.

The study followed the Preferred Reporting Items for Systematic Reviews and Meta-Analyses (PRISMA) guidelines, searching five medical databases for relevant scholarly papers. The search was limited to studies published from January 2010 to March 2024. These databases included PUBMED, Medline, Web of Science, Google Scholar, and ScienceDirect. The study assessed clinical trials, randomized controlled trials, and prospective studies involving patients with corneal ulcers. It included studies that reported outcomes such as corneal opacity density, neovascularization, and the healing period of epithelial defects. Two independent, experienced researchers screened the extracted papers for potential inclusion, and in cases of disagreement, they engaged in discussions to resolve the issue. Relevant data were extracted from the selected studies using a structured data collection form. The Cochrane risk of bias assessment scale was used to evaluate the risk of bias in clinical trials and randomized controlled trials, while the Newcastle-Ottawa Quality Assessment Scale (NOS) was applied to assess prospective/retrospective, cross-sectional, and cohort studies.

The results of the study indicate that BCLs and amniotic membrane transplantation (AMT) are safe and effective treatments for dry eye disease and corneal lesions. In cases of severe ulcerative keratitis and neurotrophic keratitis, AMT was shown to improve healing outcomes. AMT was also found to be more effective in treating deep stromal ulcers. Additionally, it enhances ocular integrity and promotes anatomical restoration following corneal perforation. Furthermore, AMT demonstrated significant efficacy in treating dry eye disease (DED). When combined with a bandage contact lens, dehydrated AMT was found to reduce symptoms of DED. However, its effectiveness remains limited in certain cases. The study also revealed that amniotic membrane suspension (AM suspension) serves as a non-invasive alternative to conventional AMT.

In conclusion, AMT was more effective in managing deep stromal ulcers and neurotrophic keratitis, especially when retained for longer durations. Bandage contact lenses were useful in managing post-surgical epithelial defects and enhancing membrane retention. Future research should investigate long-term outcomes and combined therapy protocols.

## Introduction and background

The cornea is a highly sensitive structure, and its healing process can be complex following injury. This is primarily due to the challenge of managing a wound in such a delicate area without compromising the overall integrity and function of the eye [1]. Conditions such as dry eye disease (DED), neurotrophic keratitis, and corneal ulcers can further complicate the corneal healing process [2,3]. Patients dealing with these issues face a higher risk of complications due to impaired epithelial regeneration and increased risk of scarring [2,4]. To mitigate these risks, interventions such as bandage contact lenses (BCLs) and amniotic membrane transplantation (AMT) are commonly employed. Both methods contribute to reducing the risk of adverse outcomes by facilitating faster and safer healing. Although they differ in technique and clinical application, each is effective in specific scenarios.

Bandage contact lenses (BCLs) function by forming a protective barrier over the corneal surface, shielding the epithelium and promoting its regeneration [5]. They are mostly used for treating corneal abrasions, aiding in recovery after surgery, and addressing persistent epithelial defects [6]. By acting as a buffer, BCLs protect the cornea from friction caused by eyelid movements during blinking, while also alleviating the pain and discomfort associated with corneal injuries or conditions [6]. Elsewhere, AMT involves using a piece of amniotic membrane as a graft or dressing to support corneal healing [7]. This membrane has regenerative and anti-inflammatory properties that are crucial for the healing process [7]. AMT is typically preferred for severe corneal injuries, such as deep stromal ulcers and neurotrophic keratitis, as well as for persistent epithelial defects and ocular surface reconstruction after surgery [8]. In these cases, the amniotic membrane plays a vital role in promoting epithelial growth and reducing scarring. Amniotic membrane transplantation (AMT) can be implemented in two primary ways, depending on the nature and severity of the ocular injury. One method is dressing, where the membrane acts like a temporary bandage to cover the area and help it heal. The other method is grafting, in which the membrane is used to fill in any gaps or damaged areas in the cornea or conjunctiva [9].

Despite increasing clinical use of both BCLs and AMT, previous reviews have not clearly outlined their comparative effectiveness. Moreover, there is a lack of consensus in current clinical guidelines regarding first-line use. This systematic review addresses this gap. The research draws on high-quality clinical evidence from trials, randomized controlled studies, and prospective studies to evaluate the safety and effectiveness of these two methods in promoting corneal healing.

## Review

Materials and methods

This study adopted the guidelines set forth by Preferred Reporting Items for Systematic Reviews and Meta-Analyses (PRISMA) [10]. Relevant scholarly publications from 2010 to 2024 were identified through a search of four medical databases, namely PubMed, Web of Science, Google Scholar, and ScienceDirect. Boolean operators (‘AND’, ‘OR’) and controlled vocabulary such as MeSH terms were applied to refine the search strategy. This review was not registered on PROSPERO, but adhered to PRISMA 2020 guidelines. Due to heterogeneity in study design and outcomes, data were synthesized narratively. No subgroup or sensitivity analyses were performed.

A combination of keywords was used in searching for relevant articles for review, general terms included: “bandage contact lens”, “therapeutic contact lens”, “soft contact lens”, “amniotic membrane”, “amniotic membrane transplantation”, “amniotic membrane graft”, “corneal wound healing”, “corneal regeneration”, “corneal repair”, “corneal epithelial defect”.

Inclusion and exclusion criteria

This systematic review included studies that explored, either comparatively or individually, the role of bandage contact lenses (BCLs) and amniotic membrane transplantation (AMT) in corneal wound healing. Eligible studies comprised cohort studies, randomized controlled trials (RCTs), observational studies, retrospective analyses, and longitudinal studies that investigated the effects of BCLs and/or AMT in promoting corneal epithelial regeneration, reducing inflammation, or facilitating recovery in cases such as corneal ulcers, epithelial defects, or post-surgical corneal injuries.

The primary outcomes of interest included epithelial healing time, corneal clarity, visual acuity, complication rates, pain relief, and recurrence or persistence of corneal defects. Studies comparing either intervention to standard care, to each other, or to alternative treatments were included. Only articles published in English between 2010 and 2024 were considered for inclusion. Additionally, animal-only or in vitro studies without clinical application, case reports, reviews, editorials, or studies without extractable clinical outcomes were excluded.

Eligibility, data extraction, and management

The eligibility criteria were carefully developed to ensure the inclusion of high-quality, clinically relevant studies that offer valuable insights into the individual or comparative roles of BCLs and AMT in corneal wound healing. The retrieved articles were rigorously assessed for eligibility by comparing them with the pre-defined inclusion and exclusion criteria. To maintain methodological rigor, the review process strictly adhered to PRISMA guidelines. Any discrepancies during study selection or data extraction were resolved through thorough discussion until a consensus was reached. All data relevant to the review objectives were systematically retrieved, evaluated, and documented to ensure a comprehensive and unbiased synthesis of the available evidence.

Statistical data analysis

Statistical analysis for this review was conducted using Cochrane's Review Manager (RevMan) (version 5.4.1), which was employed to calculate key metrics including risk ratios (RRs), standard errors (SEs), random effects (REs), and confidence intervals (CIs). Meta-analysis was not conducted due to the high level of clinical and methodological heterogeneity across the included studies. Laboratory studies were used to provide biological context (e.g., growth factor profiles), while clinical trials informed outcome comparisons. A random-effects model was applied to account for expected variability across studies. To evaluate heterogeneity, the I² statistic was used, with values interpreted as indicating low, moderate, or high heterogeneity depending on the percentage thresholds. Funnel plots and summary bar graphs were generated to assess potential publication bias and item-specific risk of bias. P-value<0.05 was considered statistically significant across all analyses.

Results

A total of 384 records were initially identified through database searches across PubMed, Web of Science, Google Scholar, and ScienceDirect. After removing 35 duplicates, 349 unique records were screened. During the screening phase, 339 records were excluded based on titles and abstracts that did not meet the inclusion criteria.

**Figure 1:**
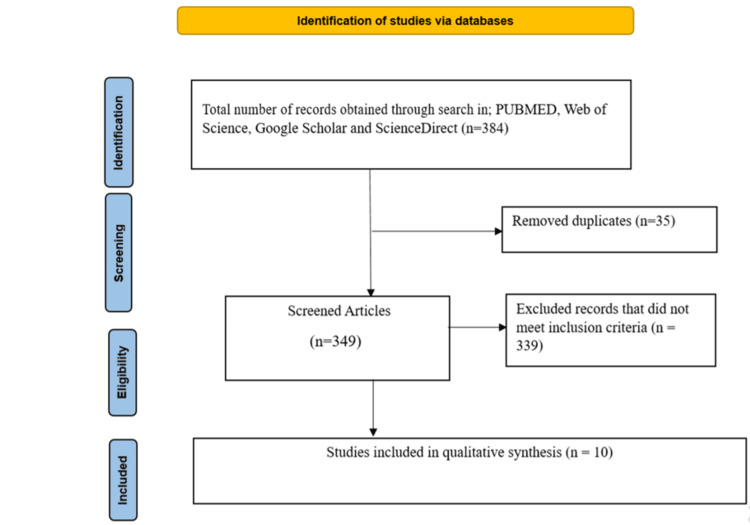
PRISMA flow diagram illustrating study selection process.

The remaining 10 full-text articles were assessed for eligibility. All 10 articles met the predefined inclusion criteria and were included in the final review. These studies were used for qualitative synthesis, focusing on the comparative and individual effectiveness of BCLs and AMT in corneal wound healing (Figure 1).

Study characteristics

The key characteristics of the studies included in this review are summarized in Table 1. All ten selected publications focused on evaluating the role of bandage contact lenses versus amniotic membrane in corneal wound healing. These studies, conducted across various global regions, provide a diverse perspective on the effectiveness of these interventions. The research designs varied, including prospective and retrospective clinical trials, randomized controlled trials, comparative studies, and experimental laboratory investigations. The sample sizes ranged from small pilot trials to large-scale retrospective case series. Additionally, all studies were published in English and adhered to rigorous methodological standards.

**Table 1:** Characteristics of included studies evaluating amniotic membrane applications in ocular surface disorders AM: Amniotic membrane; AMT: Amniotic membrane transplantation; hAMT: Human amniotic membrane transplantation; dAM: Dehydrated amniotic membrane; sBCL: Specialized bandage Contact Lens; DED: Dry eye disease; OSDI: Ocular surface disease index; SANDE: Symptom assessment in dry eye; DEQ-5: Dry eye questionnaire-5; GA: Glutaraldehyde; DAS: Dialdehyde starch; EGF: Epidermal growth factor; TGF-β1: Transforming growth factor beta 1; IGF-1: Insulin-like growth factor 1; LSCD: Limbal stem cell deficiency; FDA: Food and Drug Administration; BCVA: Best-corrected visual acuity; HCECs: Human corneal epithelial cells; MMP9: Matrix metallopeptidase 9; ELISA: Enzyme-linked immunosorbent assay; BrdU: Bromodeoxyuridine; PCNA: Proliferating cell nuclear antigen; PG: patch grafts; HGF: Hepatocyte growth factor.

Authors	Study type	Intervention	N	Outcome/Results	Conclusion
Gris et al. 2002 [11]	Prospective interventional case study	AM implantation as a therapeutic contact lens in patients with epithelial defects. Two groups: Group 1 – persistent epithelial defects; Group 2 – surgically induced defects. In some cases, a soft contact lens was applied postoperatively to support AM placement.	20	In Group 1 (persistent epithelial defects), complete epithelialization occurred in 3 of the 10 cases and partial epithelialization in 3 others. Notably, successful healing was more likely when the AM remained in place for at least 2 weeks. In contrast, early detachment (within 1 week) was associated with incomplete healing or no response. In Group 2 (surgically induced defects), all patients achieved complete epithelialization regardless of how long the membrane stayed in place. The use of a soft contact lens postoperatively in some cases helped prolong membrane retention. No intra- or postoperative complications were observed, and the mean retention time for the membrane was 12.5 days.	The study found that AM implantation used as a therapeutic contact lens is a safe and effective treatment for various epithelial defects. Longer retention of the membrane significantly improved healing outcomes in persistent epithelial defects. The combination of amniotic membrane with a postoperative soft contact lens appeared particularly beneficial in enhancing implant stability and promoting epithelialization. The authors recommend considering this approach as a surgical alternative when conventional medical treatments fail, especially as a less invasive option compared to tarsorrhaphy.
Turkoglu et al. 2013 [12]	Retrospective comparative study	Comparison of autologous serum eye drops (Group I) and AMT (Group II) in patients with neurotrophic corneal ulcers	42	In Group I, 70% of eyes healed completely, with a mean epithelialization time of 22.1 ± 8.0 days. In Group II, 72.7% of eyes healed completely, with a mean epithelialization time of 20.0 ± 4.64 days. Both groups showed visual improvement (90% in Group I and 77.2% in Group II). Persistent epithelial defects were observed in some cases, mainly those with HSV-related ulcers or deep stromal involvement. No recurrence occurred during the follow-up period (approx. 6–7 months).	Both autologous serum eye drops and AMT were effective in promoting healing in neurotrophic keratitis. However, multilayered AMT was more effective than serum drops in cases of deep stromal ulcers, particularly those due to herpes simplex virus.
Bulut et al. 2023 [13]	Retrospective cohort study	Cryopreserved human amniotic membrane transplantation (hAMT) using the overlay technique in patients with infectious ulcerative keratitis unresponsive to standard medical therapy	13	The mean epithelial healing time was 32.1 ± 14.04 days. A positive recovery response was observed in 76.9% of cases. The mean membrane residence time was 14.1 days, and the average time from initial presentation to AMT was 9.7 days. Most patients received a single-layer membrane, and the mean ulcer size was 9.7 mm. No major complications were reported, and the duration of hospitalization was reduced post-AMT.	hAMT proved to be effective in enhancing wound healing in severe infectious ulcerative keratitis, especially when performed early. The study supports that early application and prolonged membrane retention may improve clinical outcomes and reduce the duration of hospitalization.
Krysik et al. 2020 [14]	Retrospective case series	Comparison of AMT and corneal PG in surgical management of corneal melting and perforations over a 5-year period	189	Anatomical reconstruction of the anterior chamber and restoration of ocular integrity were achieved in 86% of eyes treated with AMT and 79% with PG. However, multiple interventions were often necessary: 33% of eyes required more than one surgical procedure. Visual improvement was limited, with better outcomes seen in the PG group. Postoperative complications included persistent epithelial defects (especially in neurotrophic and autoimmune cases), ocular hypertension, and, in some cases, the need for penetrating keratoplasty. Despite complications, AMT had fewer severe adverse outcomes compared to PG.	Both AMT and PG are viable techniques for managing corneal perforations. However, AMT is less invasive and preferable in early or less severe cases due to its epithelial healing properties and lower complication rate. PG provides better tectonic support in cases with structural damage or tissue exposure. A tailored approach based on underlying etiology and perforation severity is essential for optimal outcomes.
Travé-Huarte & Wolffsohn, 2024 [15]	Prospective, double-masked randomized controlled trial	Sutureless dehydrated amniotic membrane (Omnigen®) applied via a specialized bandage contact lens (OmniLenz®) vs. bandage contact lens alone, for moderate-to-severe DED	70	The dAM+sBCL group showed a 65% reduction in OSDI scores at 6 months and significantly reduced corneal dendritic cells and improved nerve parameters compared to baseline. Both treatments reduced corneal and conjunctival staining, but the dAM+sBCL group had more positive responders at 1 and 3 months in nerve and inflammation markers. The treatment was well tolerated with no serious adverse events.	A one-week application of sutureless dehydrated amniotic membrane delivered via bandage contact lens significantly improved symptoms, corneal nerve health, and inflammation markers in patients with moderate-to-severe DED. The treatment demonstrated a rapid and sustained therapeutic effect, supporting its use as a safe, non-surgical, and effective intervention.
Travé-Huarte & Wolffsohn, 2024 [16]	Prospective pre-post interventional study	Bilateral sutureless application of dehydrated amniotic membrane (Omnigen®) under a specialized bandage contact lens (OmniLenz®) in patients with moderate-to-severe dry eye disease (DED)	35	One month post-treatment, there was a 42% reduction in OSDI scores, a 31%–33% decrease in SANDE frequency and severity scores, and a 32% reduction in DEQ-5 scores. Significant improvement was also noted in lid wiper epitheliopathy width. No significant adverse effects were reported, and visual acuity remained stable.	The study demonstrates that a short-term, bilateral application of dehydrated amniotic membrane using a bandage contact lens significantly improves symptoms and some signs of DED, offering a non-invasive, effective, and well-tolerated treatment option for patients refractory to conventional therapies.
Yi et al. 2020 [17]	Experimental laboratory study with a pilot human trial	Development and evaluation of contact lens-shaped AMs using DAS and GA as crosslinking agents for sutureless ocular surface treatment	6	DAS-crosslinked AMs retained transparency, enhanced tensile strength, and maintained structural integrity better than GA-crosslinked AMs. DAS-AMs showed significantly higher growth factor retention (EGF, TGF-β1, IGF-1, etc.), better biocompatibility, and faster wound healing both in vitro and in rabbit corneal epithelial injury. In the human trial, DAS-AMs remained stable under bandage contact lenses for up to 7 days with good tolerance and no complications.	DAS-crosslinked, contact lens-shaped AMs demonstrated strong mechanical properties, excellent biocompatibility, and effective corneal wound healing, thus allowing sutureless application. They offer a promising, patient-friendly alternative to traditional AM placement, with fewer complications and potential for outpatient use.
Di Girolamo et al. 2009 [18]	Prospective pilot clinical trial	Use of FDA-approved contact lenses as a substrate and delivery device for ex vivo autologous epithelial progenitor transplantation in LSCD patients	3	All three patients achieved reestablishment of a stable and transparent corneal epithelium with no recurrence of conjunctivalization or neovascularization. Visual acuity improved in all cases, with symptom scores significantly reduced. The ocular surface remained stable throughout the follow-up period (8–13 months).	The novel technique utilizing soft contact lenses for the expansion and transplantation of autologous ocular surface progenitors proved safe and effective in restoring corneal surface integrity in LSCD patients. It presents a promising, minimally invasive, and autologous alternative to traditional grafting methods, offering flexibility and avoiding xenogeneic materials.
Eslani et al. 2019 [19]	Randomized parallel-controlled clinical trial	AMT on the entire ocular surface combined with conventional medical therapy vs. medical therapy alone in Roper-Hall grade IV ocular chemical injury	60	Corneal epithelialization time was similar between groups (72.6 ± 30.4 days in the medical group vs. 75.8 ± 29.8 days in the AMT group, P = 0.610). Final BCVA was also comparable (2.06 ± 0.67 logMAR in the medical group vs. 2.06 ± 0.57 logMAR in the AMT group, P = 0.85). Central corneal neovascularization occurred more frequently in the medical therapy group (73.3%) than the AMT group (53.3%), though this difference was not statistically significant (P = 0.108).	AMT combined with medical therapy did not significantly accelerate corneal epithelialization or improve visual acuity compared to medical therapy alone in patients with severe chemical injuries. The findings suggest that while AMT may have theoretical benefits, its impact in this context was not clinically significant.
Choi et al. 2009 [20]	In vitro experimental study	Topical AM suspension was applied to HCECs at 5% and 30% concentrations to assess proliferation and migration	N/A (cell-based)	AM suspension significantly enhanced epithelial cell migration and proliferation in a dose-dependent manner. Cells treated with 30% AM showed greater MMP9 expression and reduced E-cadherin and fibronectin expression, supporting increased motility. ELISA confirmed high levels of HGF and EGF in AM suspensions. The BrdU assay and PCNA expression indicated that 30% AM markedly increased proliferative activity compared to control.	AM suspension preserved the biochemical wound-healing properties of traditional AM transplantation. It effectively promoted corneal epithelial cell migration and proliferation, suggesting it could serve as a non-invasive, growth factor-rich topical treatment for corneal epithelial healing.

Risk of bias assessment

The quality of the included prospective studies was assessed using the Newcastle-Ottawa Quality Assessment Scale (NOS) (Table 2) [21]. Based on the scoring criteria, four of the six studies were rated as high quality with a low risk of bias [11,14,16,20], while the remaining two were classified as moderate quality with a moderate risk of bias [12,13]. Overall, the studies demonstrated a generally strong methodological quality, supporting the reliability and validity of the findings presented in this review.

**Table 2:** Newcastle Ottawa Quality Assessment Scale (NOS) results for included observational studies. A study was given up to one star (*) for each numbered item in the outcome and selection categories. Comparability, however, was rated as high as two stars. according to the evaluation's findings (**). *Demonstrates a low risk of bias in both selection and outcome. However, in terms of comparability, ** indicates a low chance of bias, whereas (-) indicates a significant risk of bias.

Author	Selection				Comparability	Outcome			Total score (1-3 high risk), (4-6 moderate risk), (7-9 low risk)
	Representativeness of the exposed cohort	Selection of the non-exposed cohort	Ascertainment of exposure	Demonstration that outcome of interest was not present at start of study	Comparability of cohorts on the basis of the design or analysis	Assessment of outcome	Was follow-up long enough for outcomes to occur	Adequacy of follow up of cohorts	
Gris et al., ^[11]^	*	-	*-	*	*	*	*	*	7/9
Turkoglu et al., ^[12]^	*	*	*	-	*	*	*	-	6/9
Eren et al., ^[13]^	*	*	*	-	*	*	*	-	6/9
Krysik et al., ^[14]^	*	*	*	*	*	*	*	*	8/9
Travé-Huarte & Wolffsohn., ^[16]^	*	-	*	*	*	*	*	*	7/9
Choi et al., ^[20]^	*	*	*	*	*	*	*	-	7/9

**Figure 2:**
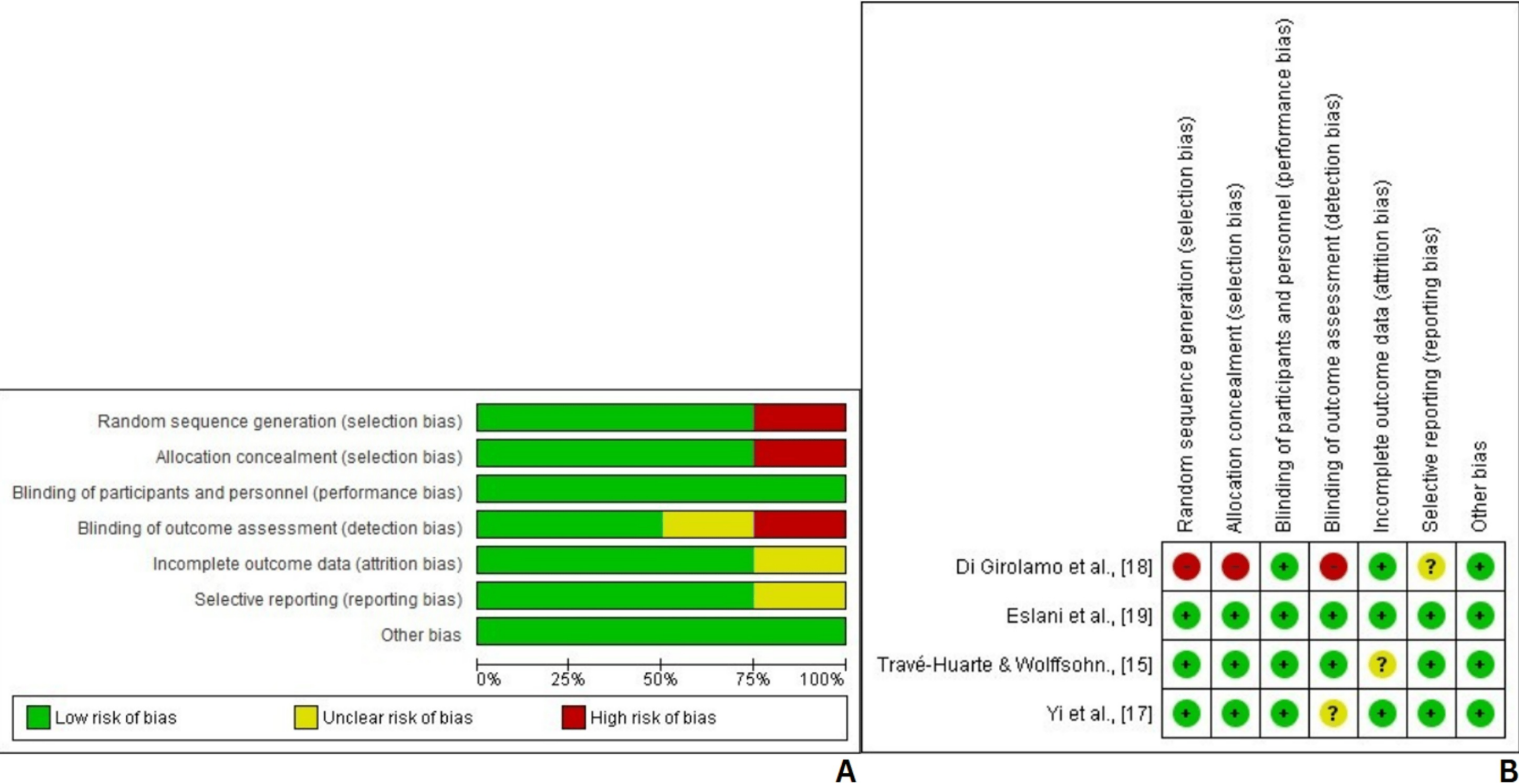
Cochrane risk of bias assessment Figure A shows the overall risk of bias summary across the included randomized controlled trials (RCTs). Figure B details study-specific assessments.

The Cochrane Risk of Bias Assessment Tool was used to evaluate the methodological quality of the included clinical and randomized controlled trials [22]. Among the four trials assessed [15,17-19], two demonstrated a low risk of bias across all seven domains, while another two showed a low risk of bias in 75% (five out of seven) of the domains. One trial exhibited a low risk of bias in 50% (three out of seven) of the domains. These findings indicate that the included studies were generally of good methodological quality. Studies with high risk in key domains were interpreted cautiously and were not used to drive major conclusions (Figure 2).

Discussion

DED is characterized by instability of the tear film and disruption of ocular surface homeostasis, leading to severe pain [11,13]. Since 2005, the prevalence of DED has been reported to be increasing over the years [11-20]. The etiology of the disease includes tear film instability, which leads to mechanical stress and epitheliopathy, as well as inflammation and damage to the ocular surface [16,17]. DED represents only one of several ocular surface conditions explored in this review. Due to the rising incidence of the disease, this systematic review focuses on presenting high-level clinical evidence supporting the effectiveness of bandage contact lenses versus amniotic membrane in corneal wound healing.

AMT consistently promoted epithelial regeneration, particularly in deep stromal ulcers and severe infected keratitis. Gris et al. reported that longer membrane retention improved outcomes, with all patients achieving epithelialization [11]. Turkoglu et al. found that multilayered AMT was more effective for HSV-induced ulcers [12]. Bulut et al. observed an average healing time of 32.1 ± 14.04 days with AMT in severe keratitis, suggesting that early application and prolonged retention may enhance outcomes [13]. Yi et al. reported that DAS-AM was biocompatible and promoted faster epithelial repair [17]. In contrast, BCLs were most effective for minor epithelial injuries and for retaining AMT post-surgery. Di Girolamo et al. highlighted BCLs’ ability to promote corneal epithelial integrity in LSCD [18].

Vision improved across most AMT-treated cases with structural healing. However, Eslani et al. noted no significant difference in final best-corrected visual acuity between AMT and medical therapy in cases of chemical injury [19]. Visual gains were also reported following BCL-assisted procedures. BCLs offered mechanical protection and reduced blink-induced shear stress, thus enhancing comfort. Travé-Huarte and Wolffsohn confirmed this in DED, where BCLs and dAM+SCL combinations significantly reduced nerve parameters and improved patient-reported outcomes (OSDI, DEQ-5, SANDE scores) [15,16]. AMT showed minimal complications but required surgical fixation. BCLs were well-tolerated with low risk, especially when used short-term or to support AM retention. Travé-Huarte et al. reported no significant side effects with dAM+BCL in DED cases [16].

Choi et al. found that AM suspension significantly enhanced the migration and proliferation of epithelial cells, with a 30% AM suspension displaying higher levels of key growth factors and improved cellular motility. This highlights the potential of AM suspension as a non-invasive, growth factor-rich topical treatment to promote corneal epithelial healing [20].

This systematic review has several limitations. One significant limitation was the heterogeneity across study designs, intervention protocols (e.g., AMT techniques), and outcome measures precluded quantitative synthesis. Another limitation was the inclusion of studies with small sample sizes and short follow-up periods. Additionally, the high risk of bias reported in some RCTs may lead to biased findings. Evaluating results is also challenging due to variations in AMT techniques, such as single-layer and multilayer applications, as well as differences in treatment regimens incorporating adjunct medications. As this review encompasses a broad range of ocular conditions and therapeutic approaches, drawing definitive conclusions regarding their effectiveness in corneal wound healing remains difficult. Nonetheless, the findings provide valuable insights that contribute to strengthening the evidence base in this field. Future research should prioritize well-designed RCTs with larger sample sizes, standardized treatment protocols, and longer follow-up periods to enhance the reliability of the conclusions.

## Conclusions

This systematic review found that AMT was effective in managing deep stromal ulcers and severe infectious keratitis. Furthermore, the review highlights that soft contact lenses can enhance AMT outcomes by stabilizing the membrane and improving comfort and epithelial healing. The study also revealed that AMT is a good way to use soft contact lenses since they improve healing and stabilize the membrane. Nevertheless, in situations of severe chemical damage, AMT had little effect on enhancing vision or accelerating epithelialization. Bandage contact lenses also promote the repair of epithelium. BCLs alone were effective in supporting epithelial repair, especially for mild epithelial defects and postoperative recovery. In order to strengthen the evidence base noted in this study. Future studies should focus on randomized controlled trials comparing standardized AMT techniques, such as single-layer versus multilayer application.

## References

[1] Mobaraki M, Abbasi R, Omidian Vandchali S, Ghaffari M, Moztarzadeh F, Mozafari M (2019). Corneal repair and regeneration: current concepts and future directions. Front Bioeng Biotechnol.

[2] Vera-Duarte GR, Jimenez-Collado D, Kahuam-López N, Ramirez-Miranda A, Graue-Hernandez EO, Navas A, Rosenblatt MI (2024). Neurotrophic keratopathy: general features and new therapies. Surv Ophthalmol.

[3] Wilson SE (2020). Corneal wound healing. Exp Eye Res.

[4] Baratta RO, Schlumpf E, Buono BJ, DeLorey S, Calkins DJ (2022). Corneal collagen as a potential therapeutic target in dry eye disease. Surv Ophthalmol.

[5] Lim L, Lim EW (2020). Therapeutic contact lenses in the treatment of corneal and ocular surface diseases-a review. Asia Pac J Ophthalmol (Phila).

[6] Chaudhary S, Ghimire D, Basu S, Agrawal V, Jacobs DS, Shanbhag SS (2023). Contact lenses in dry eye disease and associated ocular surface disorders. Indian J Ophthalmol.

[7] Sanders FW, Huang J, Alió Del Barrio JL, Hamada S, McAlinden C (2024). Amniotic membrane transplantation: structural and biological properties, tissue preparation, application and clinical indications. Eye (Lond).

[8] Meller D, Pires RTF, Mack RJS (2000). Amniotic membrane transplantation for acute chemical or thermal burns. Ophthalmology.

[9] Hofmann N, Rennekampff HO, Salz AK, Börgel M (2023). Preparation of human amniotic membrane for transplantation in different application areas. Front Transplant.

[10] Page MJ, McKenzie JE, Bossuyt PM (2021). The PRISMA 2020 statement: an updated guideline for reporting systematic reviews. BMJ.

[11] Gris O, del Campo Z, Wolley-Dod C, Güell JL, Bruix A, Calatayud M, Adán A (2002). Amniotic membrane implantation as a therapeutic contact lens for the treatment of epithelial disorders. Cornea.

[12] Turkoglu E, Celik E, Alagoz G (2014). A comparison of the efficacy of autologous serum eye drops with amniotic membrane transplantation in neurotrophic keratitis. Semin Ophthalmol.

[13] Bulut O, Musayeva G, Selver OB (2023). Impact of adjuvant amniotic membrane transplantation in infectious ulcerative keratitis. Int Ophthalmol.

[14] Krysik K, Dobrowolski D, Wylegala E, Lyssek-Boron A (2020). Amniotic membrane as a main component in treatments supporting healing and patch grafts in corneal melting and perforations. J Ophthalmol.

[15] Travé-Huarte S, Wolffsohn JS (2024). Sutureless dehydrated amniotic membrane (Omnigen) application using a specialised bandage contact lens (OmniLenz) for the treatment of dry eye disease: a 6-month randomised control trial. Medicina (Kaunas).

[16] Travé-Huarte S, Wolffsohn JS (2024). Bilateral sutureless application of human dehydrated amniotic membrane with a specialised bandage contact lens for moderate-to-severe dry eye disease: a prospective study with 1-month follow-up. Clin Ophthalmol.

[17] Yi S, Huh MI, Hong H, Yoon D, Park HS, Kim DS, Kim HK (2020). Development of contact lens-shaped crosslinked amniotic membranes for sutureless fixation in the treatment of ocular surface diseases. Transl Vis Sci Technol.

[18] Di Girolamo N, Bosch M, Zamora K, Coroneo MT, Wakefield D, Watson SL (2009). A contact lens-based technique for expansion and transplantation of autologous epithelial progenitors for ocular surface reconstruction. Transplantation.

[19] Eslani M, Baradaran-Rafii A, Cheung AY, Kurji KH, Hasani H, Djalilian AR, Holland EJ (2019). Amniotic membrane transplantation in acute severe ocular chemical injury: a randomized clinical trial. Am J Ophthalmol.

[20] Choi JA, Jin HJ, Jung S, Yang E, Choi JS, Chung SH, Joo CK (2009). Effects of amniotic membrane suspension in human corneal wound healing in vitro. Mol Vis.

[21] (2025). The Newcastle-Ottawa Scale (NOS) for assessing the quality of nonrandomised studies in meta-analyses. https://www.ohri.ca/programs/clinical_epidemiology/oxford.asp#.

[22] HigginsJPT HigginsJPT, Altman DG, Gøtzsche PC (2011). The Cochrane Collaboration’s tool for assessing risk of bias in randomised trials. BMJ.

